# Quantitative model of eukaryotic Cdk control through the Forkhead CONTROLLER

**DOI:** 10.1038/s41540-021-00187-5

**Published:** 2021-06-11

**Authors:** Matteo Barberis

**Affiliations:** 1 Systems Biology, School of Biosciences and Medicine, Faculty of Health and Medical Sciences, University of Surrey, Guildford, UK; 2 Centre for Mathematical and Computational Biology, CMCB, University of Surrey, Guildford, UK; 3 Synthetic Systems Biology and Nuclear Organization, Swammerdam Institute for Life Sciences, University of Amsterdam, Amsterdam, The Netherlands

**Keywords:** Dynamical systems, Biochemical networks

## Abstract

In budding yeast, synchronization of waves of mitotic cyclins that activate the Cdk1 kinase occur through Forkhead transcription factors. These molecules act as controllers of their sequential order and may account for the separation in time of incompatible processes. Here, a Forkhead-mediated design principle underlying the quantitative model of Cdk control is proposed for budding yeast. This design rationalizes timing of cell division, through progressive and coordinated cyclin/Cdk-mediated phosphorylation of Forkhead, and autonomous cyclin/Cdk oscillations. A “*clock unit*” incorporating this design that regulates timing of cell division is proposed for both yeast and mammals, and has a DRIVER operating the incompatible processes that is instructed by multiple CLOCKS. TIMERS determine whether the clocks are active, whereas CONTROLLERS determine how quickly the clocks shall function depending on external MODULATORS. This “*clock unit*” may coordinate temporal waves of cyclin/Cdk concentration/activity in the eukaryotic cell cycle making the driver operate the incompatible processes, at separate times.

## Introduction

Coordination of DNA replication (S-phase) and cell division (M-phase) is achieved by sequential activation of enzymatic activities that oscillate throughout the cell division cycle. These activities are realized by cyclin-dependent kinases or Cdks, formed by a catalytic (kinase) and a regulatory (cyclin) subunit. The cyclin determines the timing of Cdk activation, and a progressive activation and inactivation of a cyclin/Cdk complex is able to generate its sustained oscillations^1,2^.

**Fig. 1:**
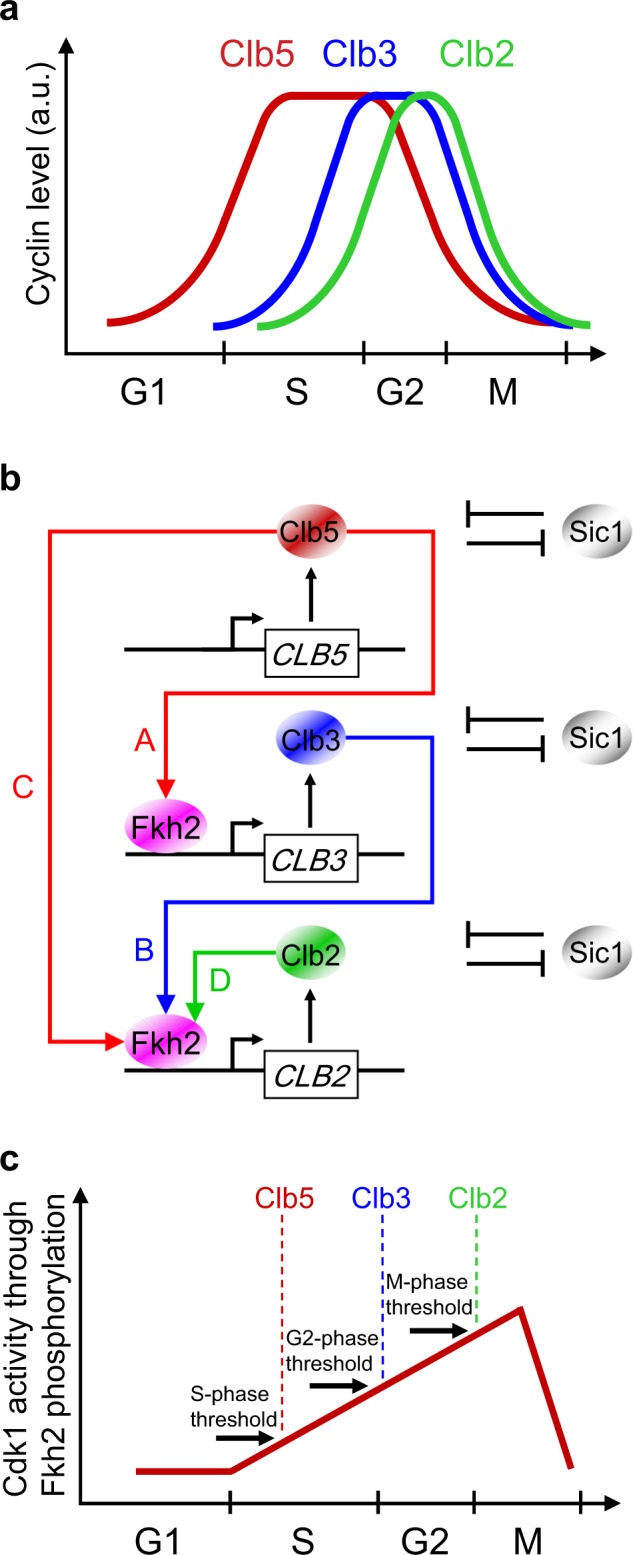
Waves of cyclins pattern for the mitotic (Clb) cyclins throughout cell cycle progression. **a** Qualitative description of alternating waves of expression of mitotic cyclins throughout the cell cycle phases. In budding yeast, Clb indicates mitotic cyclins: Clb5,6 (red color) trigger DNA replication in S-phase; Clb3,4 (blue color) trigger completion of S-phase and early mitotic events in G2 phase; Clb1,2 (green color) trigger late mitotic events and cell division in M-phase. **b** Model for the transcriptional regulation of the mitotic Clb/Cdk1 complexes. A coherent type I feed-forward loop (FFL) may synchronize activation of mitotic Clb cyclins through the Fkh2 transcription factor: Clb5/Cdk1 promotes *CLB3* transcription (arrow A), Clb3/Cdk1 promotes *CLB2* transcription (arrow B) together with Clb5/Cdk1 (arrow C), and Clb2/Cdk1 promotes *CLB2* transcription by a positive feedback loop (PFL, arrow D). For the sake of clarity, the Cdk1 subunit has been omitted. Arrows represent activating interactions among the Clb/Cdk1 complexes, whereas bar-headed black lines represent the mutual, inhibiting interactions between Clb/Cdk1 complexes and their stoichiometric inhibitor Sic1 (adapted from ref. ^8^). **c** Systems biology-driven design that rationalizes the quantitative model or “threshold model” of Cdk1 control: a progressive activation of Fkh2 is realized through a processive, multi-step phosphorylation mediated by different thresholds of Clb/Cdk1 activities determined by the accumulation of Clb cyclins (adapted from^11^).

Waves of multiple cyclin/Cdk activities raise and fall at a specific timing to guarantee cell cycle frequency, with mitotic (Clb) cyclins driving cell cycle events from S- through M-phase (Fig. 1a)^3–5^. Accumulation of cyclins occurs at definite temporal windows of transcriptional control. However, strikingly, molecular mechanisms responsible for the timely coordination of the “waves of cyclins” pattern^6,7^ remain elusive.

We have recently demonstrated how the sequential order of waves of Clb cyclins is achieved by coupling Cdk with transcriptional activities in budding yeast^8^. Through mathematical modeling, we have predicted a Clb/Cdk-mediated regulation of an activator molecule that stimulates mitotic cyclin expression^8^. This prediction was validated experimentally, identifying the Forkhead (Fkh) transcription factor Fkh2—major activator of Clb2, which regulates the timing of cell division—as a pivotal molecule responsible for the sequential activation of mitotic *CLB3* and *CLB2* genes^8^. We discovered that Clb waves are temporally synchronized by Fkh2, and that a Clb/Cdk1-mediated regulation of Fkh2 modulates *CLB* expression. Thus, integrated computational and experimental analyses point to Fkh2 as a dynamical regulator of cyclin/Cdk complexes. In control engineering, elements that are designed for dynamic systems to behave in a desired manner are indicated as “controllers.” Similarly, Fkh2 is indicated here as a CONTROLLER molecule, which results in the desired synchronization of the temporal expression of mitotic Clb waves.

The findings reveal a principle of design that coordinates waves of Clb cyclins appearance, with Cdk and Fkh transcription activities being interlocked to guarantee a timely cell cycle. Intriguingly, within this design Clb3/Cdk1-centered regulations appear to drive self-sustained Clb/Cdk1 oscillations^9^. Through an extensive computational analysis that explores the full set of activatory and inhibitory regulations able to generate oscillations, we have recently shown that a minimal yeast cell cycle network involving Clb/Cdk1 complexes and their stoichiometric inhibitor exhibits transient and sustained oscillations in the form of limit cycles^9^. Specifically, we uncovered that a Clb3/Cdk1-mediated positive feedback loop (PFL) and a linear cascade of activation of mitotic Clb/Cdk1 complexes from S- through M-phase—through Clb3/Cdk1 (Clb5 → Clb3 → Clb2)—are recurrent network motifs that yield sustained, autonomous oscillations of Clb/Cdk1 waves^9^ that capture their sequential activation and inactivation.

Our evidence suggests that a Fkh-mediated design principle underlies Cdk control in budding yeast, specifically synchronizing the “waves of cyclins” pattern. However, at present, a molecular mechanism that rationalizes the coordinated appearance of Clb/Cdk complexes in eukaryotes is not known. Here, a “*clock unit*” that incorporates the Fkh-mediated design is proposed to regulate the timing of cell division in both yeast and mammals, to coordinate DNA replication and cell division through modulation of temporal waves of cyclin/Cdk activity.

### The Fkh2/Clb3-centered design rationalizes the quantitative model of Cdk control in budding yeast

The mechanism uncovered for the progressive activation of the Fkh2 transcription factor, which activates—one after another—the mitotic Clb/Cdk1 complexes throughout the cell cycle, makes sense in the light of the well-known concept that unidirectional cell cycle progression is realized through the progressive increase in the Cdk activity^10^. This so-called “quantitative model” has been envisioned by the Nobel Prize 2001 recipient Sir Paul Nurse in 1996 and referred to as the “threshold model” by the Nobel Prize 2001 co-recipient Tim Hunt^11^, and has been subsequently demonstrated experimentally in fission yeast^12,13^, mammalian cells^14^, and budding yeast^15^. The quantitative model of Cdk control proposes that a progressive cyclin accumulation leads to an increase in the Cdk activity through different thresholds of activity that are required for a timely phosphorylation of targets^10^. Specifically, distinct thresholds of Cdk activity drive cell cycle progression through S-phase and M-phase, with M-phase requiring a higher threshold of cyclin level—thereby of Cdk activity—than S-phase^10^.

In line with the quantitative model of Cdk control, it has been shown in budding yeast that specificity of cyclins towards targets increases from G1 (Cln2) to S (Clb5) to G2 (Clb3) to M (Clb2) phase^16^. Specifically, a higher cyclin specificity in M-phase than in S-phase confers a higher Cdk activity in M-phase than in S-phase^16,17^. Moreover, inhibitory tyrosine phosphorylation of Clb/Cdk1 complexes—mediated by the Swe1 kinase—increases from S (Clb5/Cdk1) to M (Clb2/Cdk1) phase, thereby supporting their progressive activation throughout cell cycle progression^18^. In this scenario, the binding of Clb/Cdk1 complexes to Sic1—stoichiometric inhibitor of Clb/Cdk1 complexes^19,20^—protects the former from tyrosine phosphorylation, allowing accumulation of unphosphorylated kinase complexes^18^ that can promote DNA replication initiation dynamics at the G1/S transition upon Sic1 degradation^21,22^. These results are complementary to recent evidence that shed light on mechanistic details of phosphorylation events that are required to modulate targets at different thresholds of Cdk activity^23,24^.

Although the aforementioned studies support from different angles the concept underlying the quantitative model of Cdk control proposed by Sir Paul Nurse, a molecular mechanism that rationalizes the coordinated appearance of mitotic waves of Clb cyclins is currently not known. The molecular mechanism in place shall be able to temporally coordinate Clb waves such that these do appear one after another, *at different times*, and do disappear *at the same time*^4,5^.

A design principle may be proposed, which provides a mechanistic basis underlying the quantitative model of Cdk control for the budding yeast. The design explains the progressive cyclin accumulation from S- to M-phase, which leads to increased thresholds in the Cdk activity, through a coherent type I feed-forward loop (FFL) that incorporates the linear cascade (Clb5 → Clb3 → Clb2) aided by PFLs^8^ and the mutual inhibition of all Clb/Cdk1 complexes with Sic1 that we discovered^25^ (Fig. 1b). This design rationalizes the occurrence of staggered waves of Cdk1 activity and the progressive activation of Clb5, Clb3, and Clb2 mitotic cyclins—which are observed throughout a cell cycle round. Specifically, an increase in the extent of Fkh2 phosphorylation from S- to M-phase, mediated by the progressive accumulation first of Clb5/Cdk1, then of Clb3/Cdk1, and ultimately of Clb2/Cdk1 kinase activities, ensures the timely occurrence of Clb waves (Fig. 1c). An involvement of two different phosphorylation patterns mediated by various Clb/Cdk1 activities may be envisioned for the Fkh2-mediated transcription of *CLB* genes: (i) Clb5/Cdk1-mediated specific phosphorylation events on Fkh2 for *CLB3* transcription, which may be reinforced by the Clb3/Cdk1-mediated PFL on *CLB3* gene; and (ii) Clb3/Cdk1- and Clb5/Cdk1-mediated specific phosphorylation events on Fkh2 for *CLB2* transcription, which is reinforced by the Clb2/Cdk1-mediated PFL on *CLB2* gene.

The details of this sequential phosphorylation have been not yet elucidated and are currently under investigation in our laboratory. However, this hypothesis is supported by evidence from us and others that the Fkh2 phosphosites S683 and T697 are recognized by all Clb/Cdk1 kinase activities, and that their deletion leads to a reduction of Fkh2 phosphorylation^8,26^.

Many Cdk1 targets contain clusters of multiple phosphorylation sites^27^ and multisite phosphorylation of targets by cyclin/Cdk1 activities has been proposed to transform a graded protein kinase signal into an ultrasensitive switch-like response. Therefore, it can be speculated that dynamics and sequence of individual Clb/Cdk1-dependent phosphorylation events differ within the multisite phosphorylation patterns activating Fkh2, and that a potential cooperativity of the individual phosphorylation events is realized by a different specificity (binding affinity) of Clb5/Cdk1, Clb3/Cdk1, and Clb2/Cdk1 complexes to Fkh2. Thus, a mechanism of cooperativity among Clb/Cdk1-dependent phosphorylation events may promote the progressive activation of Fkh2 from S- to M-phase, to drive waves of *CLB* expression, thereby of Clb/Cdk1 waves of activity for a timely cell division, and ensure robust cell cycle oscillations.

The cooperativity that can be envisioned among Clb/Cdk1-dependent phosphorylation events on Fkh2 finds a parallel with studies that provided insights into the multisite phosphorylation mechanism that degrades Sic1^28–31^. We have shown that, similarly to Fkh2, Sic1 interacts with all Clb cyclins^32^ and parallel studies have shown that a switch-like Sic1 destruction is dependent on a complex process in which both Cln2/Cdk1 (G1 phase) and Clb5/Cdk1 (S-phase) activities act in processive multi-phosphorylation steps^29^. Multisite phosphorylation patterns can act as timing signature that modulates substrate activity at different cyclin/Cdk1 thresholds^23^ and Fkh2 may be regulated by similar cooperative phosphorylation patterns.

Clb/Cdk1-mediated phosphorylation patterns on Fkh transcription factors may control a timely gene expression through diverse mechanisms: (i) regulation of transcriptional elongation and termination^33^, (ii) regulation of a repressive chromatin structure in the coding region of *CLB2* together with chromatin-remodeling ATPases^34^, (iii) regulation of Sir2-4 silencing proteins^35^, and/or (iv) regulation of metabolic genes that are crucial for cell growth and division^36^. It is apparent that Fkh are hubs that have the ability to control gene expression by connecting intracellular pathways that operate at different but specific times. However, the coordination of these mechanisms with the staggering waves of Clb cyclins is currently unexplored.

Finally, in addition to the cyclin/Cdk1-mediated (cooperative) phosphorylation of targets, further mechanisms may be involved in the quantitative model of Cdk control to modulate the timing of target’s phosphorylation. In budding yeast, the protein phosphatase Cdc14 has been proposed to be involved in this process by imposing Cdk thresholds through antagonization of Clb2/Cdk1-mediated phosphorylation, thus contributing to the correct order of cell cycle events^37–40^. Further investigations are required to disentangle the delicate balance between Clb/Cdk1 and phosphatases in the quantitative model of Cdk control.

### A Forkhead CONTROLLER-based “*clock unit*” for cell cycle timing in yeast

Where the common view of cell division is that of a single cycle, a more sophisticated design may be recognized in it of *multiple* overlapping “oscillators” within a cycle. These oscillators are quasi-independent molecular-network “clocks” that, independently, contribute to the timing and optimal function of the cycle as a whole. Each oscillator emerges as a time-wave in the concentration of one out of a group of the regulatory cyclins.

Each CLOCK, i.e., each of the cyclins, determines the times at which molecular activities are (in)activated. It does this by binding to the catalytic Cdk, the actual DRIVER. The driver, Cdk, controls the cell cycle but not its temporal dynamics, which are instead controlled by the cyclins, the clocks. This organization repeats itself for each cell cycle phase: different cyclins progressively bind to Cdk as defined by successive waves of cyclins. The resulting cyclin/Cdk complexes define the timing of the cell cycle phase(s) in a unidirectional and irreversible manner; here, the concept of *multiple* overlapping “oscillators” within a cell cycle connects with the existing understanding of cell cycle regulation.

In budding yeast, there are nine distinct cyclins grouped in four subgroups. These subgroups, together, clock four phases of the cell cycle (these phases do not correspond precisely to the classically recognized G1, S, G2, and M, although this is often presented as a simplification). Here, the focus is on three clocks, i.e., cyclins Clb5,6 (CLOCK1), Clb3,4 (CLOCK2), and Clb1,2 (CLOCK3), their oscillations being responsible for the alternation of the incompatible processes of DNA replication, chromosome segregation, and cell division from S- through M-phase, respectively. Although the number of clocks should equal the number of functional phases, some cell cycle phases could be regulated by more than one clock and some cyclins may begin to regulate long before the beginning or ending of the phase they trigger. Of note, G1 cyclins are not part of the clocks, because the quantitative model for Cdk control has been proposed to describe the Cdk requirement for S-phase and mitosis^10,11^.

**Fig. 2:**
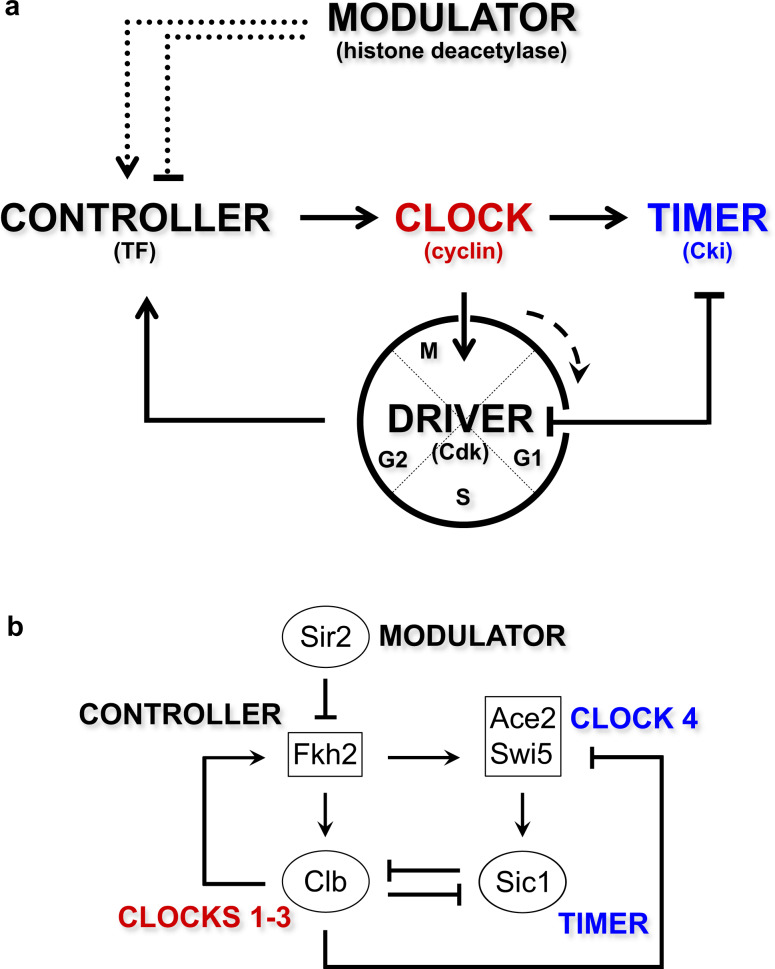
“*Clock unit*” of the budding yeast cell cycle. **a** A “*clock unit*” of the cell cycle is formed by (i) a DRIVER (Cdk1 kinase) that, together with the CLOCK (cyclin: Clb cyclin; red color), drives cell cycle events through various phases; (ii) a TIMER (Cki, cyclin-dependent kinase inhibitor: Sic1; blue color) that inhibits the DRIVER; (iii) a CONTROLLER (TF, transcription factor: Fkh2) that activates the CLOCK (cyclin: *CLB* gene); and (iv) a MODULATOR (histone deacetylase: Sir2) that modulates the activity of the CONTROLLER. **b** “*Clock unit*” that integrates CLOCKS 1–3 (Clb cyclins; red color), CONTROLLER (Fkh2 transcription factor), MODULATOR (Sir2 histone deacetylase), CLOCK4 (TF TIMER, Ace2–Swi5 transcription factors; blue color), and TIMER (Cki, Clb/Cdk1 kinase inhibitor Sic1; blue color) together with the known regulations occurring among them.

The investigations conducted in the last 20 years in our laboratory enable to propose candidate molecules that form and regulate the “*clock unit*” underlying the quantitative model of Cdk control through waves of Clb activities, i.e., of the clocks, and therewith the temporal coordination of the Clb/Cdk1 complexes: CLOCKS (Clb cyclins), DRIVER (Cdk1 kinase), TIMER (Sic1 inhibitor), CONTROLLER (Fkh2 transcription factor), and MODULATOR (Sir2 histone deacetylase) (Fig. 2a). One is (i) a TIMER of the *activity* of the clock/driver (Clb/Cdk1) complex: this is the inhibitor of the Clb/Cdk1 activity through cyclin-mediated recruitment of the Clb/Cdk1 inhibitor (Cki) Sic1^19,20^. The other two molecular mechanisms control the *concentration* waves of Clb cyclins: (ii) a CONTROLLER of transcription of each *CLB* gene promoted by the previous Clb/Cdk1 complex: this is the Fkh transcription factor^8^ and (iii) MODULATOR(S) of the activity of the Fkh transcription factor through inhibition by chromatin (epigenetic) factor(s): these are the histone deacetylases such as Sir2^41^ and Sin3/Rpd3^42^.

Of note, the Anaphase-Promoting Complex (APC)-mediated mechanism of degradation of each cyclin within a Clb/Cdk1 complex—promoted by the subsequent Clb/Cdk1 complex (see refs. ^25,43^ and references therein)—is not explicitly considered in the scheme of Fig. 2a. We and others have shown, computationally and experimentally, that this mechanism is less relevant for oscillations of the Clb/Cdk1 activity^25,44–46^, despite its relevance to modulate Cdk1 activity through abolishment of Clb levels before the start of a new cell cycle. In fact, Sic1-mediated feed-forward regulations are required to maintain an oscillation-like behavior of Clb/Cdk1 activities and to prevent mitotic cyclin synthesis^25,44^. Thus, we did propose early that Sic1, rather than Clb degradation, acts as a TIMER of the temporal waves of mitotic Clb cyclins^25^.

**Fig. 3:**
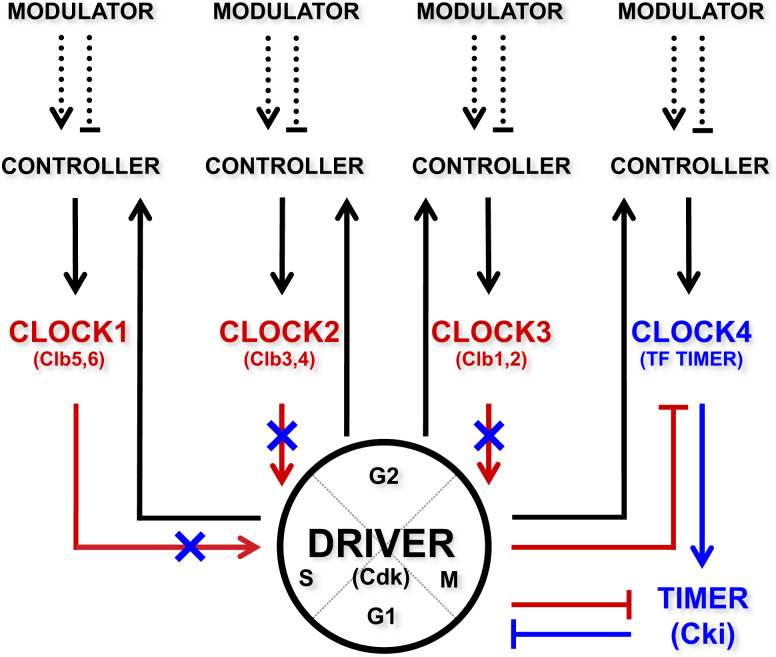
View of a dynamic cell cycle as “*clock units*”. Interaction scheme of the “*clock units*” Clb5,6 (CLOCK1), Clb3,4 (CLOCK2), and Clb1,2 (CLOCK3), which interlock one other based on the regulation core in Fig. 2a. In addition, CLOCK4 (TF TIMER, Ace2–Swi5 transcription factors) (i) is activated by the CONTROLLER, (ii) activates the TIMER (Cki), and (iii) is inhibited by the DRIVER. Red arrows and bar-headed red lines indicate CLOCKS 1–3-mediated reactions, whereas blue arrows and bar-headed blue lines indicate CLOCK4-mediated reactions. Blue crosses indicate inhibition of CLOCKS 1–3 by CLOCK4.

A design principle that can be therefore proposed has the DRIVER (Cdk1 kinase) operating the incompatible processes that is instructed by multiple CLOCKS (Clb cyclins). A TIMER (Sic1 inhibitor) determines whether the clocks are active, whereas a CONTROLLER (Fkh2 transcription factor) determines how quickly the clocks proceed depending on the external signal(s) or MODULATOR (Sir2 histone deacetylase) (Fig. 2b). This “*clock unit*” may interlock, i.e., coordinate together, the three clocks—cyclins Clb5,6 (CLOCK1), Clb3,4 (CLOCK2), and Clb1,2 (CLOCK3) (Fig. 3). Within this scenario, an additional clock may be envisioned with the DRIVER (Cdk1 kinase) and TIMER (Sic1 inhibitor) being interlocked in a fine balance between mutual activation and inhibition. A design principle proposed for this clock (CLOCK4) has the DRIVER (Cdk1) that activates the CONTROLLER (Fkh2), which in turn regulates the transcription of CLOCK4 (the *ACE2* and *SWI5* transcription factors) (Fig. 2b)^36,47–51^) that is responsible for the expression of the TIMER (Sic1)^52–54^. Beside the documented mutual inhibition of DRIVER (Cdk1) and TIMER (Sic1) at the protein level, the DRIVER (Cdk1) inhibits CLOCK4 (Ace2–Swi5)^55^, determining how quickly the timer is inactive and thereby whether the driver is active (Figs. 2b and 3).

The three mechanisms act as timers that switch each clock ON and OFF, and determine how fast each clock is progressing. The clocks are among each other’s timers. Intriguingly, this coordination is such that waves of cyclin/Cdk activity occur sequentially, *at different times*, throughout the various cell cycle phases but disappear *at the same time* at cell division, as observed experimentally^4^. Although the processes around some cyclins individually have been investigated, neither the transcriptional mechanism inter-connecting all cyclin subgroups nor how timing of the cyclin waves is managed by the eukaryotic cell are understood. This is remarkable, being this timing of obvious importance to coordinate incompatible cell cycle phases. It is therefore critical to elucidate how the coordination among CLOCKS, DRIVER, TIMER, CONTROLLER, and MODULATOR is achieved such that waves of cyclin/Cdk activity can coordinate sequentially, possibly through the progressive, increasing Clb/Cdk1-mediated phosphorylation of the CONTROLLER (Fkh transcription factor). For this coordination to occur, it is critical to determine the molecular mechanisms that control—together—the timing of the cyclin waves and how the design(s) use any of three Clb clocks to prevent overlap of incompatible processes, thus guaranteeing a timely cell cycle.

By answering the questions above, why and how the Clb/Cdk activities are switched OFF simultaneously at cell division, and not progressively with the same temporal organization as that of their activation, will be elucidated. Switching OFF simultaneously the Clb/Cdk activities suggests that the three Clb clocks shall overlap; if they would not do so, the clocks would be independent and would switch OFF progressively, one after another, with a different temporality.

Therefore, a systems biology strategy of integrating appropriate computational modeling with a quantitative experimental investigation is the key to identify regulatory designs employed to control the timing of cellular proliferation.

### A Forkhead CONTROLLER-based “*clock unit*” for cell cycle timing in mammals

A systems biology approach that integrates predictive modeling and dedicated biological experiments has proven to be pivotal, to uncover molecular mechanisms that address cellular timing, i.e., underlying the temporal regulation of mitotic (Clb) cyclins, and reveals a principle of design of cellular reproduction that may be conserved in eukaryotic organisms including humans. This design relies on cyclin/Cdk and transcription activities being interlocked to guarantee a timely completion of the cell cycle.

In humans, the question of how the temporal coordination of DNA replication and cell division occurs to prevent their overlap is unanswered. The understanding of the molecular mechanisms underlying this coordination would help to prevent an uncontrolled, enhanced cell division, which is a typical feature of human diseases such as cancer. It is thought that multiple Cdk and cyclins control the timing of this coordination by ensuring alternation with a definite temporal delay. However, the molecules involved in this process have not been pointed out yet.

One of these molecules may be p27^Kip1^, belonging to the Kip/Cip family of cyclin/Cdk inhibitors^56,57^, which binds to Cyclin E/Cdk2 (G1 phase) and to Cyclin A/Cdk2 (S-phase) that control the timing of the S-phase onset^58^. p27^Kip1^ is often mutated in human cancers^59^. On the one hand, we have shown earlier—for the first time—the structural and functional homology of the yeast Clb/Cdk1 inhibitor Sic1 to the mammalian p27^Kip1 ^^20^. Similarly to the bimodal mechanism in place for the binding of Sic1 to Clb5/Cdk1^20,60^, it was shown that p27^Kip1^ binds to Cyclin A/Cdk2 and blocks its activity through a mechanism where the inhibitor is first recognized by a hydrophobic pocket on the cyclin subunit and, subsequently, it extends on the Cdk subunit to reach and block the Cdk catalytic pocket^61,62^.

On the other hand, through computer modeling, we have provided a rationale for the role of p27^Kip1^ to set the timing of DNA replication dynamics at the S-phase onset^63^, following the same mechanism that we did propose early for Sic1^21,22^. Both yeast and mammalian studies have been inspired by experimental findings showing that the Cdk inhibitors are responsible for the activation of cyclin/Cdk complexes^64,65^, possibly translocating them from the cytoplasm to the nucleus where they exploit their function.

It is also in the nucleus where another molecule or, more precisely, a class of molecules exploit their functions. These are the Fkh, highly conserved transcription factors in eukaryotes, from yeast to human, with roles in physiological processes and diseases. Human Fkh molecules have been intensively studied due to their crucial function in cellular processes such as cell cycle regulation^66,67^, genome replication and stability^68^, aging and oxidative stress^69,70^, metabolism^71,72^, cancer^73–76^, and neurodegeneration^77^. The human Fkh family comprises 18 subfamilies^78,79^, with two of them named Forkhead box O (FoxO) and M (FoxM) being the closest functional counterpart of the budding yeast Fkh1 and Fkh2.

**Fig. 4:**
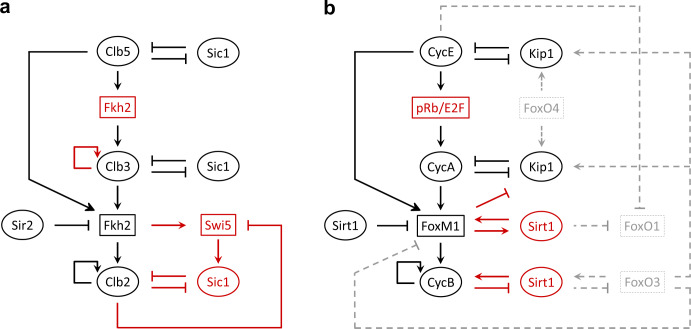
Molecular regulation that controls timing of eukaryotic cell division. **a**, **b** Comparison between the integrated “*clock units*” in budding yeast (**a**) and in mammalian cells (**b**). Homologous molecules and regulations are indicated in black color; regulations that are currently known in one organism but not in the other, and vice versa, are indicated in red color; additional regulations that occur in mammalian cells are indicated in dotted gray color (see text for details). For simplicity, the DRIVER (Cdk) has been omitted.

Although the molecular mechanism(s) that synchronizes cyclin/Cdk complexes with Fox proteins is at present unknown, the FFL + PFLs motif that it is proposed here to control the waves of mitotic Clb/Cdk1 activities in budding yeast may be transposed to the mammalian system. Intriguingly, in the latter, a number of available experimental evidence has not been connected yet in a systems view. These data can be considered—together—to speculate that the yeast “*clock unit*” (Fig. 4a, black color) may hold true also in mammalian cells (Fig. 4b, black color). In Table 1, the regulatory interactions involved in the “*clock unit*” are reported for the two organisms. Of note, the homologous of the budding yeast Ndd1 is lacking in mammalian cells.

**Table 1:** Comparable regulatory interactions involved in the “*clock unit*” of budding yeast and mammalian cells.

Budding yeast	Mammalian cells
• Clb5 (CLOCK1) and Clb3 (CLOCK2)—which can localize to the nucleus^65,86,87^—together with Cdk1 (DRIVER) may phosphorylate and activate Fkh2 (CONTROLLER), which in turn promotes expression of Clb2 (CLOCK3)^8,26^	• Cyclin E (CLOCK1) and Cyclin A (CLOCK2)—which are localized in the nucleus^88^—together with Cdk2 or Cdk1 (DRIVER) phosphorylate and activate FoxM1 (CONTROLLER1)^89–91^, which in turn promotes expression of the mitotic Cyclin B (CLOCK3)^83,92^ that is homologous to Clb2 in budding yeast
• Clb5 (CLOCK1) promotes expression of Clb3 (CLOCK2) through phosphorylation and activation of Fkh2 (CONTROLLER)^8^	• Cyclin E (CLOCK1) promotes expression of Cyclin A (CLOCK2)^93^. Of note, this activation does not occur directly but through phosphorylation and inhibition of the pRb transcriptional repressor of the E2F transcription factor, which in turn promotes expression of Cyclin A
• A PFL is in place with Clb2 (CLOCK3) promoting its own expression through phosphorylation of Fkh2 (CONTROLLER)^94^	• A PFL is in place with Cyclin B (CLOCK3) promoting its own expression through phosphorylation and activation of FoxM1 (CONTROLLER1)^95,96^
• Clb5 (CLOCK1) and Clb3 (CLOCK2) together with Cdk1 (DRIVER) are inhibited by, and inhibit Sic1 (TIMER)^19,25,29,60^	• Cyclin E (CLOCK1) and Cyclin A (CLOCK2) together with Cdk2 (DRIVER) are strongly inhibited by, and inhibit p27^Kip1^ (TIMER)^56,57,97^
• Sir2 (MODULATOR) inhibits Fkh2 (CONTROLLER)^40^	• Sirt1 (MODULATOR) inhibits FoxM1 (CONTROLLER1)^98^

Currently, unexplored regulatory interactions in budding yeast are recognized that may provide an additional level of control among the molecules forming the “*clock unit*” in mammalian cells (Figs. 4a and 4c, red color and Table 2). Furthermore, molecular mechanisms may act on top of the “*clock unit*” (Fig. 4b, dotted gray color and Table 2), to confer robustness to the mammalian complex system.

**Table 2:** Regulatory interactions that may provide additional level of control, or confer robustness to the “*clock unit*” in mammalian cells.

Additional level of control	Potentially conferring robustness
• Cyclin B (CLOCK3) together with Cdk1 (DRIVER) phosphorylates and inhibits Sirt1 (MODULATOR)^99^, with loss of activity of the latter being correlated to cell cycle withdrawal^100^	• Cyclin E (CLOCK1) together with Cdk2 (DRIVER) phosphorylates and inhibits FoxO1 (CONTROLLER2)^105^
• Sirt1 (MODULATOR) acetylates and activates Cyclin B (CLOCK3) together with Cdk1 (DRIVER)^101^	• Sirt1 (MODULATOR) acetylates and inhibits FoxO1 (CONTROLLER2)^106,107^ and FoxO3 (CONTROLLER3)^108^, although it has also been reported that Sirt1 may activate FoxO3^109,110^
• Sirt1 (MODULATOR) promotes transcription of FoxM1 (CONTROLLER1)^102^, although it has also been reported that FoxM1 promotes transcription of Sirt1^103^	• FoxO3 (CONTROLLER3) inhibits transcription of FoxM1 (CONTROLLER1)^111–113^
• FoxM1 (CONTROLLER1) inhibits transcription of p27^Kip1^ (TIMER)^104^	• FoxO3 (CONTROLLER3) and FoxO4 (CONTROLLER4) promote transcription of p27^Kip1^ (TIMER)^108,114,115^

Altogether, the evidence presented here suggests that a “*clock unit*” regulating timing of cell division may be conserved from yeast to mammalian cells. The intricacy within the network of regulations among multiple Fox proteins, Sirtuin, mitotic cyclin/Cdk complexes, and their stoichiometric inhibitor reflects possible mechanisms through which timing and robustness of cell division is ensured for the more sophisticated living organisms.

### Outlook

Deregulation of cell cycle timing, which speeds up or slows down the frequency of cyclin/Cdk oscillations, may result in disease development such as when mis-regulation of c-Myc and cyclin levels occur^80,81^. Because of the emergent role as hubs connecting intracellular pathways to control gene expression, Fkh may function as a building block that integrates regulatory modules to realize cell physiology. Within this scenario, molecular routes by which some cells (i) escape proper timing, (ii) alter dynamics of cell proliferation, and (iii) compromise viability potentially resulting in cellular dysfunctions and disease development in humans may be suggested to counter such escapes.

In humans, FoxM1 and FoxP transcription factors are the closest homologs of the yeast Fkh1 and Fkh2^67,82^, and, of note, FoxM1 regulates the expression of the mitotic Cyclin B^83^ similarly to the mechanism through which Fkh2 regulates Clb2 expression^26^. The awareness of emerging roles of FoxM1 and FoxO transcription factors as prognostic and predictive markers for the diagnosis and precise screening of cancer patients^84,85^ suggests that a multi-scale, systems biology-driven understanding of the complex regulation between cyclin/Cdk activities and Fkh-centered transcriptional network may reveal new molecular mechanisms through which these factors act in the context of human physiology and its deregulation.

## Data Availability

Data sharing not applicable to this article, as no datasets were generated or analyzed during the current study.
